# The Nucleolin Antagonist N6L Inhibits LINE1 Retrotransposon Activity in Non-Small Cell Lung Carcinoma Cells

**DOI:** 10.7150/jca.37776

**Published:** 2020-01-01

**Authors:** Kenneth S. Ramos, Sara Moore, Isabel Runge, Marco A. Tavera-Garcia, Ilaria Cascone, Jose Courty, Elsa M. Reyes-Reyes

**Affiliations:** 1 Texas A&M University College of Medicine, Department of Medicine and Institute of Biosciences and Technology, Houston, Texas 77030, USA.; 2 University of Arizona College of Medicine, Department of Medicine, Division of Pulmonary, Allergy, Critical Care, and Sleep Medicine, Tucson, Arizona 85721.; 3 University of Paris Est (UPEC), ERL-CNRS 9215, Laboratory of Growth, Reparation, and Tissue Regeneration (CRRET), UPEC, 94010 Créteil, France.; 4 Department of Cellular and Molecular Medicine, University of Arizona Cancer Center, Tucson, AZ, 85721.

**Keywords:** Nucleolin, LINE1, NSCLC, and Lung cancer.

## Abstract

Lung cancer is the most common cause of cancer death in the United States. The genome of non-small cell lung cancer (NSCLC), the most frequent lung cancer type, is strongly affected by Long Interspersed Nuclear Element (LINE1) insertions. Active LINE1s are repetitive DNA sequences that can amplify themselves in the genome utilizing a retrotransposition mechanism whereby LINE1 is copied via reverse transcription and inserted at target sites. ORF1p and ORF2p are LINE1 encoded proteins essential for LINE1 retrotransposition. LINE1s are silenced epigenetically in somatic tissues, and their reactivation has been associated with cancer pathogenesis. Here, we present evidence that nucleolin (NCL) regulates expression of LINE1-ORF1p (L1-ORF1p) in NSCLC cells. Genetic knockdown of NCL significantly inhibited expression of L1-ORF1p in various NSCLC cell lines. Treatment with the investigational NCL antagonist N6L ablated L1-ORF1p expression in all cell lines constitutively expressing L1-ORFp. N6L displayed a stronger antiproliferative activity in NSCLC tumor cell lines expressing the highest L1-ORF1p protein levels. Moreover, N6L treatment of nude mice bearing NSCLC tumor xenografts blocked L1-ORF1p expression and effectively inhibited tumor growth*.* These data indicate that L1-ORF1p expression is regulated by NCL and identify NCL as a novel promising target for pharmacological inhibition of LINE1.

## Introduction

Non-small cell lung cancer (NSCLC) is the most common type of lung cancer and the leading cause of cancer-related mortality and economic burden in the United States 1. More than 65% of NSCLC patients show cancer progression presenting with locally advanced or metastatic disease 2. Current treatments have relatively low response rates and significant toxicity. Therefore, identification of new targets and drugs is needed to aid in the development of alternative therapeutic options for patients with NSCLC 3.

LINE1s are autonomous and highly mutagenic genetic elements that mobilize throughout the genome via retrotransposition 4, 5. In humans, LINE1 copies constitute 17 to 20% of the genome, though only ~100 LINE1s per individual remain retrotransposition competent due to truncations during the course of reverse transcription and polymorphic variations in LINE1 sequence. LINE1 encodes two proteins: L1-ORF1p, a 40 kDa protein with nucleic acid binding activity, and ORF2p, a 150 kDa protein with endonuclease and reverse transcriptase activities 6. A complete cycle of L1 retrotransposition consists of transcription of L1 RNA, export into the cytoplasm, translation of ORF1 and ORF2, association of L1 RNA with ORF1 and ORF2 proteins to form ribonucleoprotein (RNP) particles, import of RNPs into the nucleus, reverse transcription, and integration into new genomic locations 7. Active LINE1s are a source of endogenous mutagenesis, with reactivation in somatic cells associated with a variety of genetic alternations including, aberrant splicing, exon skipping, gene fusions, and genome rearrangements that change gene expression and promote genomic instability 6.

The genome of NSCLCs is strongly affected by L1 insertions 8, 9. As such, systematic investigation of mechanisms responsible for the regulation of LINE1 protein expression and the specific roles of LINE1s in NSCLC progression are crucial to the identification of novel molecular targets for NSCLC therapy.

Nucleolin (NCL) is an RNA-binding protein, and more than 90% of NCL is localized in the nucleolus. However, NCL is also present in other cellular compartments, including the nucleoplasm, cytoplasm, and cell surface. NCL has multiple roles in ribosome biogenesis, transcription, DNA and RNA metabolism, DNA repair, and apoptosis 10-13. In contrast to normal tissue, NCL excessively accumulates in the cytoplasm and the cell surface (extranuclear) of several cancer cell types, including lung cancer cells 14-17. One key function of NCL is the modulation of cellular protein levels by binding mRNA targets to regulate RNA turnover and translation. NCL was recently reported to bind LINE1 RNA 18, but the functional implications of this interaction are not yet fully understood. Here, we present evidence that NCL modulates LINE1 activity by regulating the expression of L1-ORF1 protein. This interaction can be targeted pharmacologically by NCL antagonists, thus opening the door to novel therapies for lung cancer treatment.

## Methods and Materials

### Materials

NucAnt 6L (N6L) was synthesized as previously described and dissolved in 5% D‐mannitol 19. AS1411 (5'-GGTGGTGGTGGTTGTGGTGGTGGTGG-3') oligodeoxynucleotide in the desalted form was purchased from Life Technologies (Grand Island, NY). Anti-rabbit IgG and anti-mouse IgG antibodies linked to horseradish peroxidase (HRP), anti-nucleolin monoclonal (MS-3), and anti-GAPDH monoclonal antibody were from Santa Cruz Biotech. A custom made polyclonal anti-human L1-ORF1p antibody was produced by New England Peptide LLC. The antigen peptide “MGKKQNRKTGNSKTQ” sequence used to generate the rabbit polyclonal anti-human L1-ORF1p does not match the murine ORF1p amino acid sequence. Further, the specificity of the antibody against human L1-ORF1p was validated using several criteria including a single band of the expected molecular weight by Western blot, use of positive and negative control cell lines and tissue sources, specific knockdown of signal intensity using siRNAs, and high reproducibility between experimental runs and antibody lots. Biochemical validation of the anti-L1-ORF1p has been described previously 20.

### Cell cultures and treatments

Cell lines were purchased from the American Type Culture Collection (ATCC). Cells were grown in a humidified environment at 37^o^C with 5% CO_2_. Cell lines were confirmed to be free of mycoplasma contamination (MycoAlert; Lonza). NSCLC cell lines (NCI-H460, NCI-H520, NCI-H1299, and A549) were grown in RPMI medium supplemented with fetal bovine serum at a concentration of 10%, 62.5 mg/mL penicillin and 100 mg/mL streptomycin (Life Technologies). The human bronchial epithelial cell lines BEAS-2B and BZR were grown in LHC-9 medium (Thermo Fisher Scientific). Verification of all cell line identities was performed by short tandem repeat (STR) sequencing using reference databases from ATCC (Genetics Core, University of Arizona, AZ). Cells were plated 1-day before treatments and challenged with the desired concentrations of N6L or AS1411 as indicated in figure legends. For biochemical analyses, cells were lysed with buffer containing 150 mmol/L NaCl, 2 mmol/L EDTA, 50 mmol/L Tris-HCl, 0.25% deoxycholic acid, 1% IGEPAL CA-630 (pH 7.5), supplemented with protease and phosphatase inhibitor cocktails (EMD Millipore) for 5 min at 4°C, and then cleared by centrifugation at 16,000 × g for 10 minutes at 4°C. All protein concentrations were determined using the bicinchoninic acid assay (Thermo Fisher Scientific).

### Western blot analyses

Total cell lysates were resolved by SDS‐polyacrylamide gel electrophoresis and electrotransferred onto polyvinylidene fluoride membranes (Millipore) in Tris-glycine buffer containing 20% methanol. Proteins were detected by immunoblotting. Membranes were stripped of bound antibodies using Restore™ PLUS Western Blot Stripping Buffer (Thermo Fisher Scientific) and reprobed as described in figure legends.

### Nucleolin knockdown expression

NCL small interfering RNA (siRNA) duplex sequences [5'-GGUCGUCAUACCUCAGAAGtt (NCL#1, ID#144014), 5'-CGGUGAAAUUGAUGGAAAUtt (NCL#2, ID#144016)] and scrambled siRNA sequences (Silencer Negative Control #1 siRNA (AM4635)) were chemically synthesized and annealed by Life Technologies. BLAST analysis showed no homology to any sequence in the Human Genome Database, other than the intended target. siRNAs were transfected using Lipofectamine RNAiMAX (Life Technologies), according to manufacturer's directions. Briefly, cells were plated and incubated overnight to allow adherence, then transfected with siRNAs for 8 h. Cell medium was replaced with fresh complete medium, and cells incubated for 72 h before analysis as described in figure legends.

### Cell proliferation

Measurements of cell proliferation were completed using a 3-(4,5-dimethylthiazol-2-yl)-2,5-diphenyltetrazolium bromide (MTT) assay 21. This assay detects metabolic activity based on NAD(P)H-dependent cellular oxidoreductase activity and has been used as a reliable measure of cell proliferation in cultured bronchial epithelial cells 20. Briefly, 3,000 cells were seeded in quadruplicate 96-well plates and allowed to adhere overnight. Cells were treated with different concentrations of N6L and incubated for 72 h without changing the culture medium. The signal corresponding to medium from plates with no cells was subtracted as background. Cell proliferation was determined by normalizing to the proliferation of untreated cells for each cell type.

### Measurements of cell viability

BEAS-2B and H520 cells (60,000) were plated in 12-well plates one day before treatment. Cultures were either challenged with 10 μM N6L or an equivalent volume of vehicle. Adherent and floating cells were harvested after three days and pelleted by centrifugation. The pellet was resuspended in 0.4 % trypan blue/PBS solution and stained cells counted to measure viability.

### *In vivo* studies

The Institutional Animal Care and Use Committee (IACUC) at the University of Arizona approved all experimental procedures involving animals. Healthy male, weanling nude mice (Fox1nu) were purchased from Charles River Laboratories Inc. After acclimation for a week in the animal facility, mice were injected subcutaneously with a single cell suspension consisting of 3 X 10^6^ NCI-H520 cells in 200 μL PBS into each flank. When subcutaneous tumors reached a volume of approximately 100 mm^3^, the mice were randomized into two groups of 6 mice per group. The control group was given PBS and compared to animals given 10 mg/kg/day N6L dissolved in PBS three times per week by intraperitoneal injection. Tumor volume and body weights were recorded every two or three days for 16 days. Tumors were resected following euthanasia and processed for detection of L1-ORF1p expression by immunoblotting.

### Statistical analysis

Experimental replicates were independent and performed on separate days. Comparisons between treated and control groups were carried out using multiple paired two-tailed t-tests or ANOVA followed by Tukey's multiple comparisons test as specified in figure legends. Statistical significance was denoted by p-values less than 0.05.

## Results

### NCL regulates expression of LINE1

Previous reports indicate that 50% of NSCLC have increased L1-ORF1p expression across a panel of different human lung neoplasms 9. We have reported that stable ectopic overexpression of LINE1 in non-malignant human bronchial epithelial BEAS-2B cells induces oncogenic transformation and tumorigenesis *in vivo,* independent of its reverse transcriptase activity and active cycles of retrotransposition 20, 22. These findings suggest that LINE1 is involved in lung carcinogenesis and possibly serve as a novel candidate for targeted therapeutics during malignant progression of NSCLCs.

**Figure 1:**
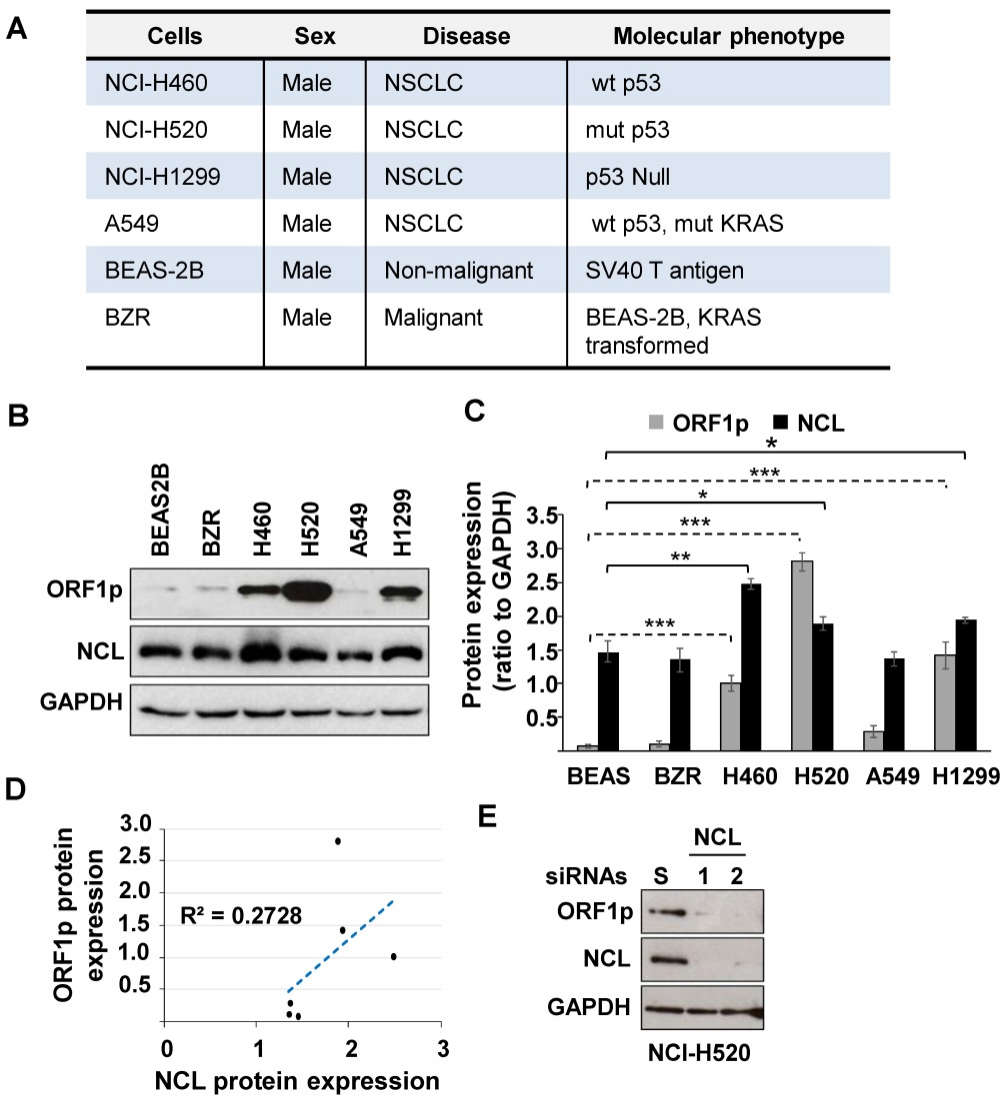
** (A)** Phenotypic profiles of lung cells employed in this study. **(B)** Whole-cell extracts from BEAS-2B, BZR, NCI-H460, NCI-H520, A549, or NCI-H1299 were examined by immunoblotting using L1-ORF1p, NCL, and GAPDH antibodies. **(C)** NCL, L1-ORF1p, and GAPDH were quantified by densitometry. Relative protein expression was expressed as NCL/GAPDH and L1-ORF1p/GAPDH ratios from three independent analyses. Error bars represent SEM. Statistical significance was determined using multiple paired two-tailed t-tests; n=3; *p < 0.05; ** p < 0.001, ** p < 0.0001. **(D)** Correlation between L1-ORF1p and NCL protein levels.** (E)** NCI-H520 cells were transfected with two different NCL siRNAs (NCL #1 and 2). Three days post-transfection, cells were analyzed for expression of L1-ORF1p, NCL, and GAPDH.

NCL modulates cellular protein levels by binding mRNA targets to control RNA turnover and translation. This protein is of interest given its ability to regulate cancer cell phenotypes and to partner with LINE1 RNA 18. Therefore, studies were conducted to determine whether NCL modulates L1-ORF1p expression in NSCLC. We first examined the relative expression of L1-ORF1p and NCL in four NSCLC cell lines (NCI-H460, NCI-H520, NCI-H1299, and A549), compared to the non-malignant BEAS-2B cell line and its ras-transformed counterpart, BZR cells (Fig. 1A). Immunoblotting analysis showed that L1-ORF1p was strongly expressed constitutively in three NSCLC cell lines (NCI-H520>NCI-H1299>NCI-H460), while relative L1-ORF1p expression was detectable at low levels in BEAS-2B, BZR, and A549 cells (Fig. 1B and C). All tested cell lines showed strong expression of NCL (Fig. 1B and C). While the expression of L1-ORF1p did not consistently correlate with NCL expression (Fig. 1D), higher levels of NCL expression were preferentially observed in NSCLC cell lines with higher LINE1-ORFp1 expression (Fig. 1C).

Next, we examined whether NCL played a role in the regulation of LINE1 by examining the effect of genetic knocking down of NCL on L1-ORF1p expression in NCI-H520 cells. Immunoblot analyses confirmed that NCL expression could be reduced by >90% in cells transfected with two distinct NCL siRNAs compared with cells transfected with scrambled siRNA (Fig. 1D). Knockdown of NCL elicited a dramatic decrease in L1-ORF1p expression (Fig 1E). These results indicate NCL is a positive regulator of L1-ORF1p expression.

### N6L, a NCL antagonist, inhibits L1-ORF1p expression

**Figure 2:**
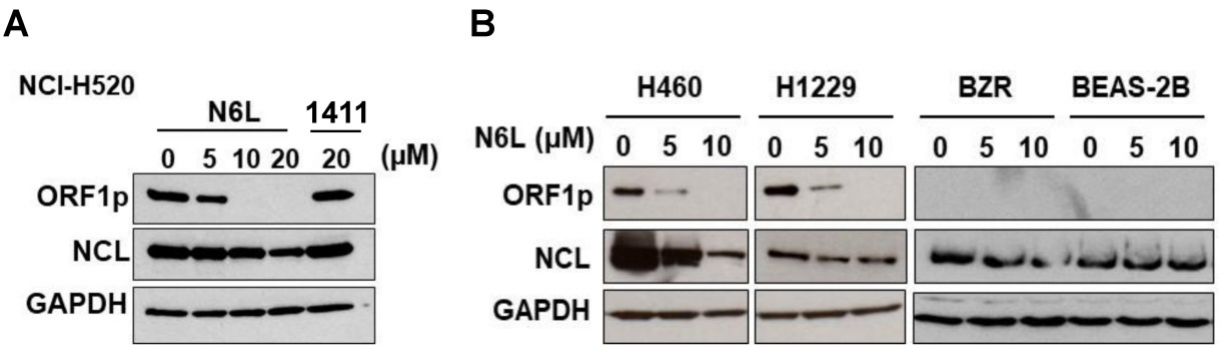
** (A)** NCI-H520 cells were treated with vehicle (5% D‐mannitol solution) or different concentration of N6L or 20 µM AS1411 (AS) for 48h. **(B)** NCI-H460, NCI-H1299, BZR, and BEAS-2B cells were treated with different concentration of N6L for 48 h. Whole-cell lysates were analyzed by immunoblotting using L1-ORF1p, NCL, and GAPDH antibodies.

To further evaluate the influence of NCL on L1-ORF1p expression, the next set of experiments was designed to determine if pharmacological agents that block NCL functions modulate expression of L1-ORF1p in NSCLC cells. Currently, N6L, a pseudopeptide, and AS141, a DNA aptamer, are the best options to study the biological functions of NCL. We analyzed L1-ORF1p expression profiles in NSCLC cells challenged with various concentrations of N6L and AS1411. N6L completely inhibited L1-ORF1p protein expression in NCI-H520, NCI-H460, and NCI-H1229 cells treated with 10 µM for 24 h (Fig. 2A and B). In contrast, AS1411 was without effect in any of the cell lines (data not shown) or in NCI-H520 cells at high concentrations (20 µM) (Fig. 2A). Because LINE1 can be reactivated under stress conditions, we tested the effects of N6L in low expressing BEAS-2B and BZR cells. N6L was without effect in these lines. These data indicate that L1-ORF1p expression is blocked by the NCL antagonist N6L and suggest that NCL may be a reasonable pharmacological target to inhibit LINE1-mediated progression of NSCLCs.

### N6L activity correlates with L1-ORF1p expression

**Figure 3:**
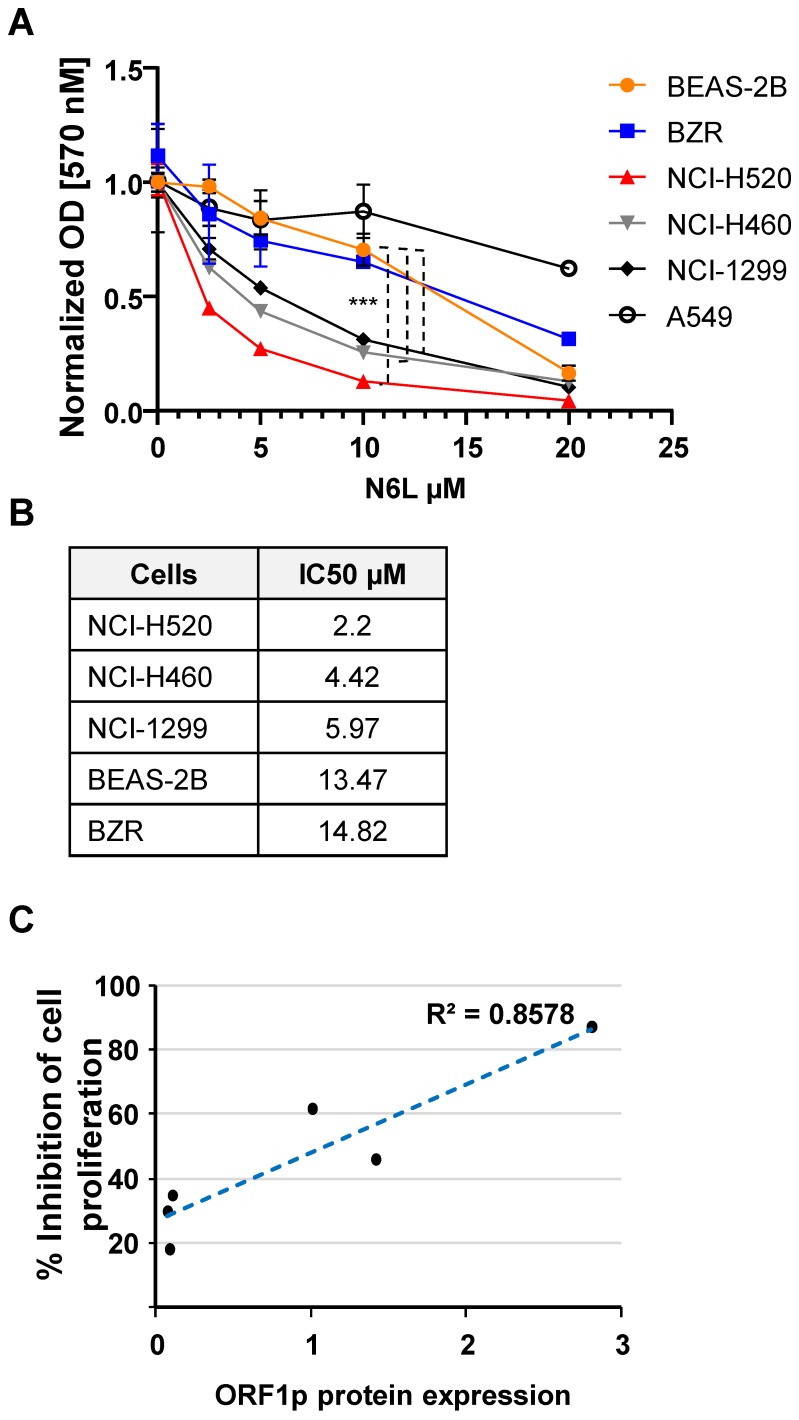
** (A)** Lung cells were treated with vehicle (5% D‐mannitol solution) or various concentrations of N6L for 3 days. The MTT assay was used to measure cell proliferation. Cell proliferation was determined by normalizing to the proliferation of untreated cells for each cell type. Error bars represent the SEM. Statistical significance was determined at 10 µM N6L comparing BEAS 2B versus H520, NCI-H460 and NCI-H1299 by ANOVA followed by Tukey's multiple comparisons test; n=4 ***p < 0.0001 **(B)** The half-maximal inhibitory concentration (IC50) of N6L was calculated after treatment for 72 h. **(C)** Correlatio n between proliferation and L1-ORF1p protein levels.

N6L has potent growth inhibitory effects in several cell lines or primary cells derived from brain, breast, prostate, T lymphocytes, colon, pancreas, skin, and kidney carcinomas 23-25. However, the effect of N6L on NSCLC cell growth has not being examined. Using the MTT assay as an indirect measure of cell proliferation we found that N6L preferentially inhibited NSCLC lines with the highest constitutive expression of L1-ORF1p (NCI-H520>NCI-H460>NCI-H1299) compared to lines with lower expression (BEAS-2B, BZR and A549 cell lines) (Fig. 3A and B).

N6L (10 µM) did not increase Trypan blue uptake in BEAS-2B or NCI-H520 cells after treatment for three days, indicating that NCL inhibition is associated with cytostatic effects, in the absence of cytotoxicity (not shown). Moreover, the antiproliferative activity of N6L correlated with the relative abundance of L1-ORF1p protein (Fig. 3C). These results suggest that the pharmacological effectiveness of N6L involves interference with L1-ORF1p expression.

### N6L inhibits L1-ORF1p expression *in vivo*

**Figure 4:**
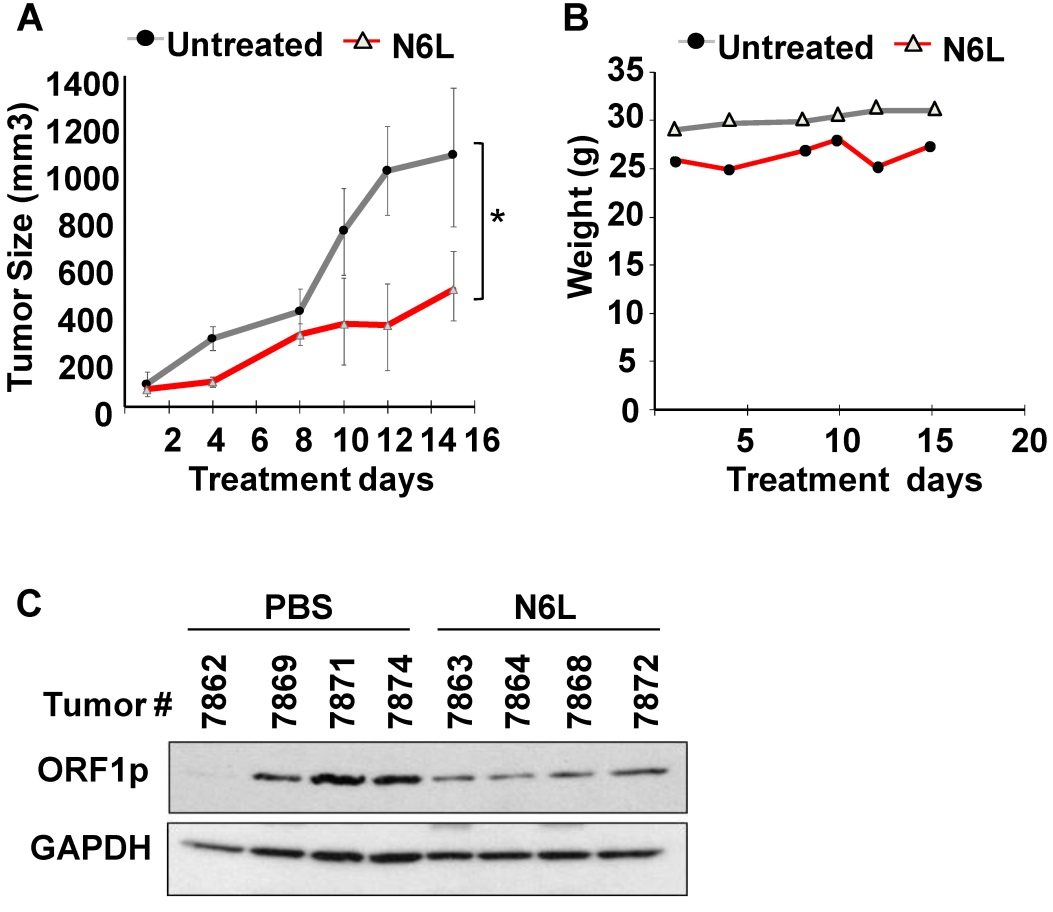
Subcutaneous tumors were established after injection of 3 X10^6^ NCI-H520 cells into 5-week old male Nu/Nu mice. Mice were randomized into two groups and treated with either PBS or 10 mg/kg/day N6L in PBS three times per week given by intraperitoneal injection. **(A)** Tumor volume; **(B)** Body weights;** (C)** LINE-1 expression in tumor tissue measured by immunoblotting using L1-ORF1p, and GAPDH antibodies. Statistical significance (*p =0.038) was determined by ANOVA followed by Tukey's multiple comparisons test. Error bars represent the SEM.

Given the inherent limitations of *in vitro* models, we next set out to determine whether the pharmacological efficacy of N6L was observed *in vivo.* To this end, nude mice with subcutaneous NCI-H520 xenografts received intraperitoneal injections of 250 μg of N6L in 200 µL PBS per day three times a week for two consecutive weeks to approximate a cumulative dose of 10 mg/kg/day. Additional control groups received an equivalent amount of vehicle alone. Tumor volumes and body weights were recorded at regular intervals. Figure 4A shows that N6L significantly inhibited tumor growth in the nude mouse xenograft model. No evidence of toxicity was observed for any of the treatments, as evidenced by the absence of changes in body weight (Fig. 4B), animal behavior, or organ examination at necropsy. Further, immunoblotting analysis showed that tumors from mice treated with N6L exhibited lower expression of L1-ORF1p compared to tumors from vehicle treated mice (Fig. 4C). These data indicate that the NCL antagonist N6L blocks LINE1 activity and tumor growth *in vivo*.

## Discussion

Evidence is presented here that NCL positively regulates the expression of L1-ORF1p in NSCLC cell lines and that NCL may be targeted pharmacologically by N6L to regulate the oncogenic activity of LINE1 in NSCLC cell lines. L1-ORF1p is expressed constitutively in NSCLC tumors but not in healthy tissues 8, 9. In keeping with previous reports, we observed that L1-ORF1p was barely detected in non-malignant human bronchial epithelium cells BEAS-2B, but significantly increased under constituve conditions in the human NSCLS tumor cell lines, H460, H520, and H1299. Interestingly, the H460, H520, and H1299 cell lines also expressed higher levels of NCL than the non-malignant human bronchial epithelium cells BEAS-2B. NCL also increases murine L1-ORF2 expression by binding to its RNA in the internal ribosome entry site (IRES) to facilitate translation 26. Consequently, NCL depletion decreases mouse L1 retrotransposition activity *in vitro* 26. These findings support the conclusion that NCL can regulate oncogenic activity by influencing the expression and relative abundance of LINE1 proteins.

NCL plays significant roles in many physiological processes such as cellular proliferation, survival, and apoptosis 27. Disruption of NCL homeostatic functions impairs cancer progression by altering signaling pathways through genetic and epigenetic mechanisms that control proliferation, survival, and metastasis of cancer cells 13. Recently, NCL was shown to form a complex with LINE1 RNA and KAP1 (a transcriptional co-repressor). This complex promotes the self-renewal of embryonic stem cells by suppressing Dux expression, a master activator of the developmental transition program in two-cell embryos 18. Thus, NCL may induce cancer progression by promoting cancer cell stemness via LINE1 RNA. Our previous findings showed that overexpression of LINE1 in non-malignant human bronchial epithelium cells induce oncogenic transformation and tumorigenesis *in vivo* 22. Our present findings showing that NCL regulates L1-ORF1p expression suggests that dysregulation of NCL functions may promote lung carcinogenesis via LINE1 activation.

N6L is a pseudopeptide that functions as a NCL antagonist through selective binding to NCL to inhibit tumor growth and angiogenesis 19, 23, 25, 28, 29. N6L blocks survival signaling pathways, slows down cell cycle progression, induces autophagy, and inhibits tumor cell invasion 24, 25, 30, 31. Given that LINE1 increases cancer cell proliferation and tumorigenesis 20, 32, the abilty of N6L to decrease L1-ORF1p expression in NSCLC cell lines and *in vivo* is consistent with observed impairments of NSCLC xenograft tumor growth. We conclude that the impact of N6L on tumor growth involves decreases in L1-ORF1p which in turn influence LINE1 oncogenic functions.

The finding that N6L, but not AS1411, blocked L1-ORF1p expression suggests that LINE1 interference by these agents is exerted via different mechanisms. N6L specifically binds to cell surface NCL to promote internalization through an endocytic mechanism. After internalization, N6L can be localized in the cytoplasm, nucleoplasm, and nucleolus 19, and is known to target both the nuclear and extranuclear functions of NCL 23, 24, 30, 33. In contrast, AS1411 is a DNA aptamer that mainly internalizes into cells through the gulp of large vesicles using the micropinocytosis endocytic mechanism. Internalized AS1411 is localized only to the cytoplasm and never localizes to the nuclei of viable cells 34. Thus, AS1411 only targets NCL extranuclear functions such as shuttling, signal transduction, or mRNA stability, which could also affect protein expression 35. The observation that AS1411 did not affect L1-ORF1p expression suggests that nuclear functions of NCL are the ones involved in regulation of L1-ORF1p expression. It has been suggested that the antiproliferative activity of AS1411 is not specific for nucleolin targeting 36. Thus, it is possible that AS1411 does not target key extranuclear functions of NCL linked to L1-ORF1p expression.

In summary, the ability of N6L to block the expression of L1-ORF1p suggests that LINE1 activity during NSCLC progression may be effectively regulated by pharmacological agents and amenable to precision therapeutics. N6L has already been tested in human clinical trials in other cancers, and clinical development efforts are ongoing. Therefore, it would be interesting to test whether N6L may be an effective therapeutic agent for NSCLC and whether the L1-ORF1p expression may serve as a biomarker to select patients for future *N6L* treatment.
